# Editorial: Steroids and the brain, volume II

**DOI:** 10.3389/fendo.2025.1556800

**Published:** 2025-01-31

**Authors:** Takayoshi Ubuka, Vance L. Trudeau

**Affiliations:** ^1^ Initiative for Research and Development, International Cancer Laboratory Co., Ltd., Tokyo, Japan; ^2^ Department of Biology, University of Ottawa, Ottawa, ON, Canada

**Keywords:** autism, depression, stress, inflammation, reproduction

Steroids are lipophilic chemicals that are composed of the perhydrocyclopentanophenanthrene four ring stricture. Steroid hormones are synthesized in the gonads, adrenals, other endocrine glands and nervous tissues, and exert various effects. Steroids and their nuclear and membrane receptors play significant roles in broad functions of the brain, such as regulation of reproduction, stress, socio-sexual behavior, aggression, cognition, mood, emotion, learning and memory.

The brain is not only the target of steroid hormones produced in the periphery but is a major site of *de novo* synthesis and catalysis (1–3). Malfunction or chemical disruption of steroid signaling is related to a variety of mental disorders such as gender dysphoria, anxiety, depression, autism spectrum disorder, cancer, and aging-related disorders such as Alzheimer’s disease (4, 5).

Following the first volume of Steroids and the Brain (6), this Research Topic has assembled basic and advanced knowledge in various aspects of steroid functions in the brain, ranging from evolutionary and physiological viewpoints to their involvement in neuropathological conditions. The articles include the role of steroids in autism, depression, stress, inflammation, and reproduction.

Autism spectrum disorder (ASD) is a neurodevelopmental disorder that is characterized by impaired communication, low interest, and repetitive behaviors. Both genetic and environmental factors are reported to play important roles in the etiology of ASD. In the USA, for example, the incidence of ASD is 1/36 among 8-year-old children (7). The 4-6:1 male to female ratio in the incidence of ASD (8), implicates sex steroids in such sex differences. Wang et al. conducted a systematic review with meta-analysis to summarize blood, urine, or saliva androgen levels in ASD individuals. It was found that androgen levels were significantly higher in ASD individuals compared to control. Subgroup analyses performed by age, sex, sample source, and measurement method showed significantly elevated levels of urinary total testosterone, urinary dehydroepiandrosterone, and free testosterone in ASD individuals. In particular, dehydroepiandrosterone levels were significantly elevated in ASD males.

Depression is more common in people with epilepsy. In a study that used the Zung Self-Reported Depression Scale, depression occurred in 26.9% of patients with epilepsy compared with 9.7% in the control cohort (9). Women with epilepsy face particular challenges with seizures and anti-seizure medications (ASMs) (10). Ogunjimi et al. investigated the association of hormones related to reproduction, including sex steroids, ASM, and depression among epileptic women. Blood samples were collected during the luteal phase (LP) and follicular phase (FP). There were statistical differences between cases and controls in testosterone and prolactin. Testosterone, FP follicle-stimulating hormone (FSH), FP estradiol, LP FSH, LP progesterone, and LP prolactin were associated with depression. Differences in various hormonal levels in epileptic women were also found by using different ASMs, such as carbamazepine and levetiracetam.

Why are there sex differences in the responses to stressors? Previous studies characterized the roles of androgens in mediating the sex differences in neuroendocrine and behavioral stress responses (11). Amaya et al. investigated whether glucocorticoid signaling in the brain may be modulated by androgens. They compared the expression of glucocorticoid receptor (GR) target genes in the brain regions where GR and androgen receptor (AR) are co-expressed after chronic treatment with corticosterone, dihydrotestosterone, combination of both, or corticosterone in combination with the AR antagonist enzalutamide. Their results showed that androgens affected glucocorticoid signaling only in the prefrontal cortex and the substantia nigra, and not in the hypothalamus, hippocampus, and ventral tegmental area. This study highlights the role of the prefrontal cortex and the substantia nigra in mediating the sex differences in the responses to stressors.

Inflammation in the brain and periphery have recently been recognized as playing critical roles in the development and progression of neurological and psychiatric disorders (12). Neurosteroids such as pregnenolone and allopregnanolone emerged as regulators of inflammatory and neuroinflammatory responses (13). Previous studies demonstrated the inhibitory effect of allopregnanolone on the activation of inflammatory toll-like receptor 4 (TLR4) signaling in RAW264.7 macrophages and the brain of alcohol-preferring rats. Balan et al. investigated the impact of allopregnanolone on the levels of interleukin-10, an anti-inflammatory mediator cytokine, and activation of the TRIF-dependent endosomal TLR4 pathway. Their results demonstrate allopregnanolone enhancement of the endosomal TRL4-TRIF anti-inflammatory signals and elevations of interleukin-10 in the male alcohol-preferring rat brain but not in females.

The rate limiting step for the synthesis of the estrogens involves aromatase (Cyp19a1) that respectively converts the androgens androstenedione and testosterone into the bioactive estrogens, estrone and estradiol. Teleosts, the bony fishes, have two paralogous aromatase genes, *cyp19a1a* and *cyp19a1b*, that are highly expressed in the ovary and the brain, respectively. It was recently shown that *cyp19a1b* mutant female zebrafish that have significantly lower estradiol levels in the brain also have altered female sexual behavior (14). Although brain aromatase is constitutively expressed in neurons in mice, *cyp19a1b* is exclusively expressed in radial glial cells in teleosts. Shaw et al. investigated the mechanistic pathways of *cyp19a1b* mutant female zebrafish in the disruption of female reproductive behavior. They found that delayed oviposition in female mutant *cyp19a1b^-/-^* zebrafish is linked to impaired arginine vasopressin (also known and vasotocin in teleosts) signaling in the brain. This study also suggests that female behavioral phenotype of *cyp19a1b^-/-^* zebrafish is a consequence of impaired processing of arginine vasopressin-dependent social cues important for mate identification and assessment.

Together, Volume I and Volume II “Steroids and the Brain” offer a broad perspective on the diverse roles of steroids in the normal vertebrate brain, under stressful conditions, or their associations with numerous neurological disorders. The emergence of methods designed for neuron-specific genetic modifications of steroidogenic enzymes and steroid receptors, coupled with single-cell transcriptomics and proteomics lays the foundation for future studies that will expand the role of steroids, and may provide novel routes for therapy.
